# Cardiomyocyte stromal interaction molecule 1 is a key regulator of Ca^2+^‐dependent kinase and phosphatase activity in the mouse heart

**DOI:** 10.14814/phy2.15177

**Published:** 2022-02-18

**Authors:** Helen E. Collins, Joshua C. Anderson, Adam R. Wende, John C. Chatham

**Affiliations:** ^1^ Division of Environmental Medicine Department of Medicine University of Louisville Louisville Kentucky USA; ^2^ Department of Radiation Oncology University of Alabama at Birmingham Birmingham Alabama USA; ^3^ Division of Molecular and Cellular Pathology Department of Pathology University of Alabama at Birmingham Birmingham Alabama USA

**Keywords:** calcium‐dependent, cardiomyocytes, kinases, protein kinase C (PKC), protein kinase G (PKG), store‐operated calcium entry (SOCE), stromal interaction molecule 1 (STIM1)

## Abstract

Stromal interaction molecule 1 (STIM1) is a major regulator of store‐operated calcium entry in non‐excitable cells. Recent studies have suggested that STIM1 plays a role in pathological hypertrophy; however, the physiological role of STIM1 in the heart is not well understood. We have shown that mice with a cardiomyocyte deletion of STIM1 (^cr^STIM1^−/−^) develop ER stress, mitochondrial, and metabolic abnormalities, and dilated cardiomyopathy. However, the specific signaling pathways and kinases regulated by STIM1 are largely unknown. Therefore, we used a discovery‐based kinomics approach to identify kinases differentially regulated by STIM1. Twelve‐week male control and ^cr^STIM1^−/−^ mice were injected with saline or phenylephrine (PE, 15 mg/kg, s.c, 15 min), and hearts obtained for analysis of the Serine/threonine kinome. Primary analysis was performed using BioNavigator 6.0 (PamGene), using scoring from the Kinexus PhosphoNET database and GeneGo network modeling, and confirmed using standard immunoblotting. Kinomics revealed significantly lower PKG and protein kinase C (PKC) signaling in the hearts of the ^cr^STIM1^−/−^ in comparison to control hearts, confirmed by immunoblotting for the calcium‐dependent PKC isoform PKCα and its downstream target MARCKS. Similar reductions in ^cr^STIM1^−/−^ hearts were found for the kinases: MEK1/2, AMPK, and PDPK1, and in the activity of the Ca^2+^‐dependent phosphatase, calcineurin. Electrocardiogram analysis also revealed that ^cr^STIM1^−/−^ mice have significantly lower HR and prolonged QT interval. In conclusion, we have shown several calcium‐dependent kinases and phosphatases are regulated by STIM1 in the adult mouse heart. This has important implications in understanding how STIM1 contributes to the regulation of cardiac physiology and pathophysiology.

## Graphical abstract

Little is known of the physiological role of stromal interaction molecule 1 (STIM1) in the heart and the signaling pathways it regulates. Here, we show that hearts lacking cardiomyocyte STIM1 have reduced activities of several calcium‐dependent kinases and phosphatases, which impact cardiac electrophysiology. Collectively, these results suggest a novel role for STIM1 in regulating cardiac homeostasis.

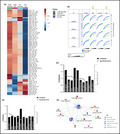

## 1 INTRODUCTION

Store‐operated Ca^2+^ entry (SOCE) is the primary pathway for voltage‐independent Ca^2+^ entry in non‐excitable cells. SOCE occurs in response to ER/SR Ca^2+^ depletion and is mediated primarily through the interaction of the ER/SR Ca^2+^ sensor, stromal interaction molecule 1 (STIM1), and plasma membrane Orai channels (Williams et al., 2001; Zhu‐Mauldin et al., 2012). Despite the fact that both STIM1 and Orai proteins are expressed in the adult heart (Williams et al., 2001; Zhu‐Mauldin et al., 2012), apart from its putative role in the progression of pathological hypertrophy (Hulot et al., 2011; Luo et al., 2012; Ohba et al., 2009; Voelkers et al., 2010), the physiological role of STIM1 in adult cardiomyocytes is poorly understood.

We have previously reported that mice with cardiomyocyte‐restricted deletion of STIM1 (^cr^STIM1^−/−^) exhibit progressive cardiomyopathy associated with ER stress (Collins et al., 2014), mitochondrial dysfunction (Collins et al., 2014), and metabolic dysfunction (Collins et al., 2018). In addition, others have shown that cardiomyocyte STIM1 may also play a role in regulating SR ER/Ca^2+^ stores through direct association with SERCA and phospholamban (Zhao et al., 2015). Furthermore, STIM1 itself may help regulate the automaticity of the sinoatrial node and therefore contribute to cardiac pacemaking (Zhang et al., 2015) and STIM1 has been shown to contribute to protection from arrhythmogenic activity (Cacheux et al., 2019). Collectively, these findings highlight the fundamental importance of STIM1 in regulating normal cardiomyocyte function; however, much remains to be regarding about the underlying signaling pathways that are regulated by STIM1.

It is well known that STIM1 is a phosphorylatable‐protein (Manji et al., 2000) and there is a growing list of kinases associated with STIM1 phosphorylation. For example, ERK1/2 phosphorylation is required for activation of SOCE (Lee et al., 2012; Pozo‐Guisado et al., 2010, 2013; Pozo‐Guisado & Martin‐Romero, 2013), and cyclin‐dependent kinase 1 (CDK1) has been implicated in the inactivation of SOCE (Smyth et al., 2009). AMPK (Lang et al., 2012), protein kinase A (PKA; Thompson & Shuttleworth, 2015), protein kinase C (PKC; Steinberg, 2012), proline‐rich kinase 2 (Yazbeck et al., 2017) and dual‐specificity tyrosine phosphorylation‐regulated kinase (Yazbeck et al., 2017) have all been reported to phosphorylate STIM1, regulating different aspects of its function. However, even though many kinases including AMPK (Lang et al., 2012), PKCα/β (Steinberg, 2012), PKA, PKG, phosphoinositide‐dependent protein kinase 1 (PDPK1), as well as protein phosphatases such as calcineurin, are Ca^2+^‐dependent (Huang, 1989; Park et al., 2013), little is known about the specific Ca^2+^ signaling pathways involved in their regulation. Therefore, we used our established ^cr^STIM1^−/−^ mouse model, combined with a discovery‐based kinomic analysis, to determine which kinases are differentially regulated by STIM1 and the potential downstream physiological consequences. The goal of this study was to also determine whether we could identify any early stage (i.e., at 12‐weeks) indications of more subtle remodeling in the regulation of signaling and kinase activity. Specifically, in the present study, we show that lack of STIM1 in cardiomyocytes results in a reduction in the phosphorylation of key Ca^2+^‐dependent kinases (PKC/PKG) and phosphatases that are associated with changes in cardiac electrophysiology. Our results demonstrate for the first time that in the absence of overt dysfunction and remodeling, STIM1 deletion leads to significant changes in the kinome, identifying potential novel STIM1‐dependent regulation of kinases that may lead to the identification of novel physiological roles of STIM1.

## 2 MATERIALS AND METHODS

### 2.1 Materials

Unless otherwise stated, all reagents and chemicals were obtained from *Fisher Scientific*.

### 2.2 Cardiomyocyte‐specific STIM1^−/−^ mice

All experimental protocols utilizing mice in this study were approved by the University of Alabama at Birmingham (UAB) Institutional Animal Care and Use Committee and adhered to the National Institutes of Health's Guide for the Care and Use of Laboratory Animals (NIH publication no. 85‐23, revised 1996). All animals received standard chow and water on an ad libitum basis, and lighting was maintained on a 12:12‐h light‐dark cycle. Cardiomyocyte‐specific STIM1^−/−^ mice (^cr^STIM1^−/−^) were generated and their respective genotypes characterized, as described previously (Collins et al., 2014, 2018). Briefly, we previously showed that ^cr^STIM1^−/−^ mice develop contractile and metabolic dysfunction from 20‐weeks of age, by 36 weeks of age have an established dilated cardiomyopathy, and by 50 weeks exhibited over 50% mortality occurring prior to the age at which we observe cre toxicity (Collins et al., 2014, 2018). Twelve‐week littermate control (STIM1^flox/flox^; αMHC‐Cre^−/−^) and constitutive, cardiomyocyte‐specific STIM1‐KO mice (STIM1^flox/flox^; αMHC‐Cre^+/−^‐ referred to as ^cr^STIM1^−/−^) male mice were used in all the following studies. This age was chosen as it is prior to the development of any evidence of cardiac pathology or decline in cardiac function (Collins et al., 2014) as well as prior to any significant changes in cardiac metabolism (Collins et al., 2018).

### 2.3 Acute subcutaneous phenylephrine treatment

^cr^STIM1^−/−^ and littermate control male mice were injected subcutaneously with either sterile saline solution or phenylephrine (PE; 15 mg/kg; Sigma P6126), as described previously (van Berlo et al., 2011). Briefly, mice injected with saline and PE were either euthanized and hearts were snap‐frozen for subsequent kinomic and immunoblot analysis 15 min following treatment or used for electrocardiogram (ECG) studies (see below). PE, an α1‐adrenergic receptor agonist, has been shown to activate SOCE (Hunton et al., 2002) and induce ERK1/2 phosphorylation (van Berlo et al., 2011). The dose of 15 mg/kg PE and treatment time of 15 min were both selected due to previous reports demonstrating a robust increase in ERK1/2 phosphorylation in wild‐type mice (van Berlo et al., 2011).

### 2.4 Analysis of the serine/threonine kinome of saline and PE‐treated hearts

**FIGURE 1:**
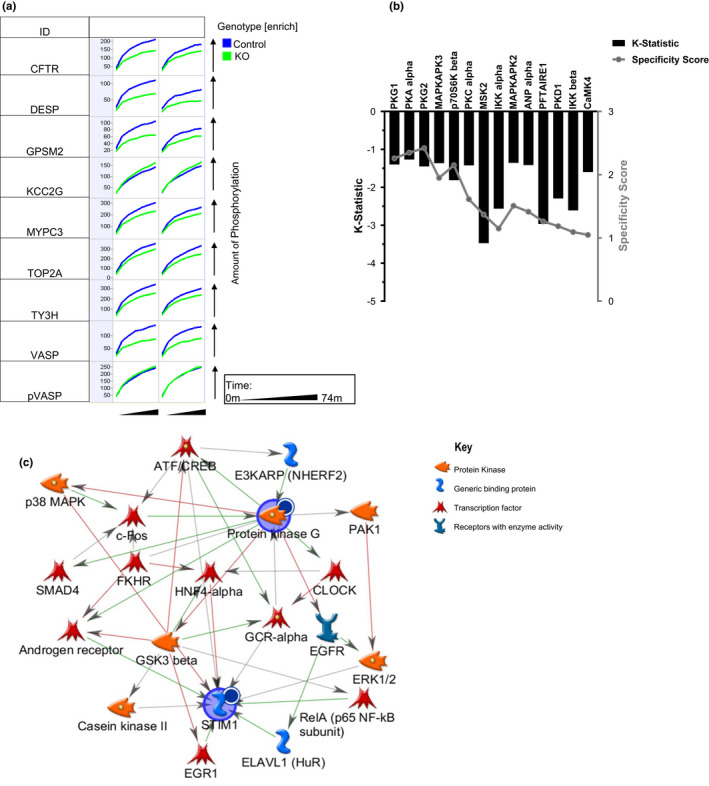
Serine/threonine kinome of control and ^cr^STIM1^−/−^ mice with saline treatment. Whole‐cell lysates of 2 μg from hearts extracted from control and ^cr^STIM1^−/−^ mice subjected to subcutaneous in vivo saline treatment were loaded onto a serine/threonine kinome chip. Kinetic phosphorylation was visualized using BioNavigator 6.0 (PamGene) with additional analysis utilizing the PhosphoNET database and MetaCore. (a) Kinetic phosphorylation (*x*‐axis per cell) over time (*y*‐axis) of altered kinases between control and ^cr^STIM1^−/−^ in response to saline treatment for selected peptides is displayed with control hearts (blue) overlaid with KO hearts (green); (b) Bar graph of kinases identified as reduced in ^cr^STIM1^−/−^ versus control using the UpKin PamApp (BioNavigator) based on kinase scores from PhosphoNET. Kinases are scored on the left y‐axis by mean kinase statistic (k‐statistic) and on the right *y*‐axis by mean specificity score; (c) MetaCore network mapping of KO altered kinases (PKG isoforms) at baseline to an overlaid STIM1 node. A Dark circle inset to the upper right of a protein indicates decreased signal in KO. Arrowheads denote literature annotated directions of interactions with the type of interaction indicated by arrow color (green‐positive; red‐negative). Legend is located on the right‐hand side of the figure panel. STIM1, stromal interaction molecule 1

Kinomic analysis of control and ^cr^STIM1^−/−^ hearts treated with either saline or PE (15 min; 15 mg/kg) was performed at the UAB kinome core facility. The PamStation 12 (PamGene international) kinomic array platform was used to assess kinomic changes in samples. Whole‐cell lysates of 2 μg were prepared, premixed, and loaded on to a high content microarray containing 12–15 amino acid targets with kinase‐phosphorylatable serine/threonine residues (STK kinome chip). Quality control experiments showed that all samples had shown to have acceptable levels of basal kinomic activity with multi‐directional changes, as seen in Figure 1a. Primary kinomic analysis was performed using BioNavigator 6.0 (PamGene). Peptides with acceptable kinetic curves and signals were used to identify upstream kinases. Altered kinases were uploaded by Uniprot ID and mapped to literature‐derived biological networks using GeneGo (www.portal.genego.com). Confirmation of specific protein/peptide targets and kinases was performed using standard immunoblotting techniques, as described below.

### 2.5 Electrocardiogram analysis

Control and ^cr^STIM1^−/−^ mice treated for 15 min with either saline or PE (15 mg/kg, s.c., 15‐min treatment) were anaesthetized by nose cone with continuous 2% isoflurane/100% oxygen mix and placed onto a monitoring platform and restrained for the duration of the recordings. Three removable color‐coded recording electrodes were sub‐dermally inserted into both upper limbs and the left leg of each mouse. The recording electrodes were connected to an ix 228/S data acquisition unit (iWorx systems. Inc). Measurements were taken at baseline and throughout duration of either saline or PE treatments. ECG data were analyzed using Labchart 8 software (AD instruments). Selected ECG cycles were averaged and data were collected on the following parameters: P, Q, R, S, and T amplitudes, RR, PR, JT, QT, P start, P peak, P end, QRS start, QRS max, QRS end, ST height, T peak, and T end were tabulated. Three‐dimensional waveform analysis was prepared from these tabulated parameters. The Labchart software was pre‐programmed for rodent ECG and four beats were averaged for calculations, as previously described (Kain et al., 2018). The Bazett formula was used to calculate QTc.

### 2.6 Calcineurin activity assay

Calcineurin activity was determined using a calcineurin cellular activity assay kit (Enzo Life Sciences, BML‐AK816), as per the manufacturer's protocol. The assay was used to determine phosphate release (total phosphatase activity), calcineurin activity (PP2B), and non‐calcineurin phosphatase activity (PP1, PP2A, PP2C) between left ventricular lysates obtained from control and ^cr^STIM1^−/−^ mice following s.c. injection of saline or PE (15 mg/kg).

### 2.7 Western blotting

Whole heart tissue was homogenized in lysis buffer (20 mM HEPES, 1.5 mM MgCl_2_, 20 mM KCl, 20% glycerol, 0.2 mM EGTA, 1% Triton X‐100, 2 mM Na_3_VO_4_, 10 mM NaF, and 2% protease inhibitor, pH 7.9). Protein was quantified using a Bio‐Rad DC assay and lysates prepared using 6× sample buffer (0.5 M Tris‐HCl, 10% SDS, 30% glycerol, 0.2% β‐mercaptoethanol, 0.012% bromophenol blue) and boiled for 5 min. Protein (10–50 µg) was separated on 7.5%, 10%, or 12% SDS–PAGE gels and subsequently transferred to Millipore immobilon polyvinylidene difluoride (PVDF) membrane. PVDF membranes were blocked for 1 h at room temperature with either 5% nonfat dry milk/TBST, 5% bovine serum albumin (BSA)/tris‐buffered saline with tween (TBST), or 2% BSA/TBST and incubated overnight at 4°C with primary antibodies specific for the following phospho‐specific proteins: phospho‐AMPKα (Thr172; Cell signaling #2535; 1:1000), phospho‐PDPK1 (Ser241; Cell Signaling #3061; 1:1000), phospho‐PKCα (Thr497; Abcam #Ab76016; 1:1000), phospho‐MARCKS (Ser152/156; R&D systems PPS010; 1:1000), phospho‐MEK1/2 (Ser217/221; Cell signaling #9121; 1:1000), and phospho‐ERK1/2 (Thr202/Tyr204; Cell signaling #4370;1:1000). In addition, membranes were incubated with primary antibodies specific for total AMPKα (Cell signaling #2532; 1:1000), PDPK1 (Cell signaling #3062; 1:1000), PKCα (Abcam #Ab192779), MEK1/2 (Cell signaling #9122), ERK1/2 (Cell signaling #4695; 1:1000), and calcineurin (Cell signaling #2614; 1:1000). After initial primary antibody exposure, membranes were washed in TBST and incubated with the appropriate HRP‐conjugated secondary antibodies (goat anti‐rabbit IgG [170‐6515; BioRad) and goat anti‐mouse [170‐6516; BioRad; 1:2000–1:5000]) for 2 h at 4°C. Blots were exposed to ECL chemiluminescence detection and images obtained using autoradiograph film. Expression was normalized to the loading control, Glyceraldehyde 3‐phosphate dehydrogenase (GAPDH) (1:5000, Abcam, Ab8245), or specific total protein levels. ImageJ (NIH) was used to perform densitometric quantification of immunoblotting data, where the ratio of target protein expression to loading control expression or phosphorylated target protein expression to total target expression were quantified.

### 2.8 Statistical analyses

Statistical significance was calculated using an ANOVA followed by a Tukey's post hoc test or Student's *t*‐test using Graph Pad Prism 8, as appropriate, whereby *p* values of <0.05 were deemed statistically significant. All data are represented as mean ± SEM. The present studies was performed using a minimum of three mice per experiment, typically between three and six mice per manipulation. Where possible, researchers were blinded to treatments and conditions. Densitometric analysis of western blotting data were performed using NIH ImageJ Software. The heatmap was generated using Metaboanalyst 4.0 software.

For kinase identification upstream of peptide targets, kinase scores derived from Kinexus (www.phosphonet.ca) for each phosphorylatable peptide residue, and residues with 90% homology were downloaded and kinases with Phosphonet V2 scores greater than 300 were rank‐ordered, with the top 12 retained. In vitro identified peptide targets of kinases on‐chip from PamGene's proprietary database were also listed to each kinase list and were given a rank order of 0. For each kinase (ALK) a difference between experimental groups (*τ*; normalized kinase statistic) is calculated. The sample mean ${\bar{p}}_{\mathrm{ij}}$ and variance $s_{\mathrm{ij}}^{2}$ of peptide *i* in each comparative group are used.

$$\tau_{\text{ALK}}=\frac{1}{n}\sum_{i=1}^{n=9}\frac{{\bar{p}}_{i1}‐{\bar{p}}_{i2}}{\sqrt{s_{i1}^{2}+s_{i2}^{2}}}.$$

A significance score is based on permutations of samples and measures how much T depends on experimental grouping of the samples, and a specificity score is based on permutation of peptides and measures how much *τ* depends on the peptide to kinase mapping. The combined overall, or mean final score, is the sum of significance and specificity.

## 3 RESULTS

### 3.1 Effect of STIM1 knockout on the cardiac kinome

All samples included in kinome analyses showed acceptable levels of kinase activity. For example, Figure 1a shows the phosphorylation time course for selected peptides that were altered between control and ^cr^STIM1^−/−^ hearts at baseline (i.e., following saline treatment). This phosphorylation time course specifically shows that phosphorylation of seven peptides was increased in control hearts at baseline. The peptides were derived from cystic fibrosis transmembrane conductance regulator (CTFR), desmoplakin (DESP), G‐protein‐signaling modulator 2 (GPSM2), myosin‐binding protein C (MYPC3), DNA topoisomerase 2A (TOP2A), tyrosine 3‐monooxygenase (TY3H), and vasodilator‐stimulated phosphoprotein (VASP). One peptide had increased phosphorylation in the KO hearts that which derived from calcium/calmodulin‐dependent protein kinase type II gamma (KCC2G). Next, we performed upstream kinase analysis to determine which kinases were giving rise to these changes in peptide phosphorylation. This analysis is presented as a plot of mean kinase statistic (k‐statistic) and mean specificity score for predicted peptide targets with mean final scores of >1.5, with decreased kinase activities in KO relative to controls as indicated on the horizontal axis (Figure 1b). As can be seen, there was an overall decrease in kinase activity in the KO group, relative to the control group. The predicted kinases that exhibited the largest decreases in the KO group included protein kinase G 1 (PKG1), protein kinase G 2 (PKG2), PKA, MAPK‐activated protein 2 (MAPKAPK2), MAPK‐activated protein kinase 3 (MAPKAPK3), natriuretic peptide A (ANP), calcium/calmodulin‐dependent protein kinase 4 (CAMK4), polycystin 1 (PKD1), inhibitor of nuclear factor‐kappa b kinase subunit (IKK) alpha and beta, mitogen and stress‐activated kinase 2 (MSK2), and S6 kinase (p70S6K). Subsequent network mapping of the altered kinases in the ^cr^STIM1^−/−^ hearts is shown in Figure 1c and shows the network of altered kinases when PKG, which exhibited the largest change in the ^cr^STIM1^−/−^ hearts, was mapped with an overlaid STIM1 node. As expected from previous reports, network analysis linked STIM1 with changes in ERK1/2 (Pozo‐Guisado et al., 2010, 2013; Pozo‐Guisado & Martin‐Romero, 2013), NF‐ΚB (Liu et al., 2016), EGR (Ritchie et al., 2011), and GSK3β (Benard et al., 2016); however analyses also showed associations of STIM1 with casein kinase II (Ck2), ELAV‐like RNA binding protein 1 (ELAVL1, also known as HuR), androgen receptor, and the glucocorticoid receptor alpha (GCRα). The complex network of changes resulting from the loss of STIM1 specifically driven by PKG highlights the potential role of STIM1 in the regulation of numerous kinases and transcription factors.

**FIGURE 2:**
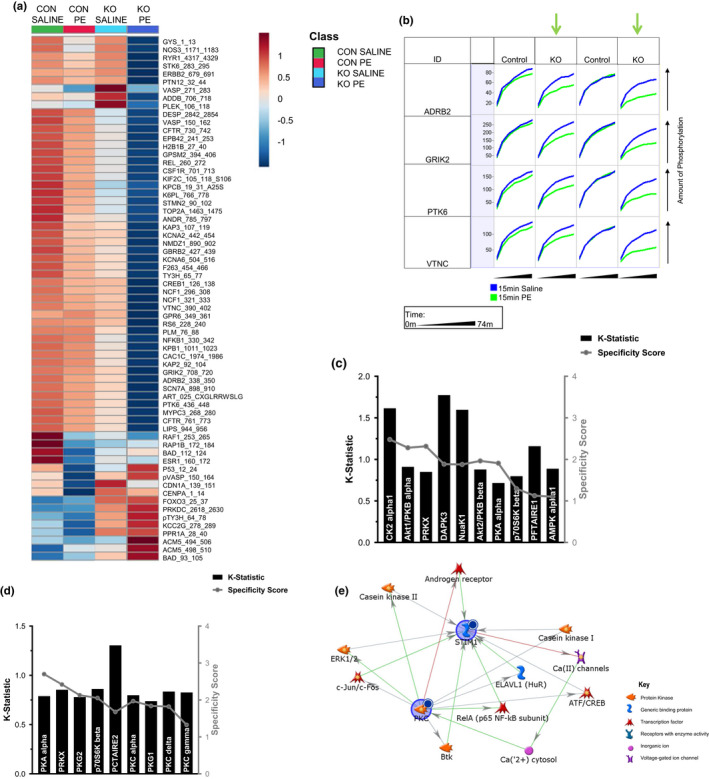
Serine/threonine kinome of control and ^cr^STIM1^−/−^ mice after 15 min phenylephrine (PE) treatment. Whole‐cell lysates of 2 μg from hearts extracted from control and ^cr^STIM1^−/−^ mice subjected to subcutaneous in vivo PE treatment (15 mg/kg) were loaded onto a serine/threonine kinome chip. Kinetic phosphorylation was visualized using BioNavigator 6.0 (PamGene) with additional analysis utilizing the PhosphoNET database and MetaCore. (a) Top 75 differentially regulated (upregulated and downregulated) peptide targets identified in control and ^cr^STIM1^−/−^ hearts treated with saline and PE were arranged into a heatmap using MetaboAnalyst software; (b) Kinetic phosphorylation (*x*‐axis per cell) over time (*y*‐axis) of altered kinases between control and ^cr^STIM1^−/−^ in response to PE. Green arrows highlight the four selected peptides that were differentially altered by PE in KO hearts; (c) Bar graph of altered kinases in control hearts treated with PE upstream of array peptides; (d) Bar graph of altered kinases in ^cr^STIM1^−/−^ hearts treated with PE upstream of array peptides. Kinase prediction is performed using the UpKin PamApp (BioNavigator) based on kinase scores from PhosphoNET. Kinases are scored on the left *y*‐axis by mean kinase statistic (k‐statistic) and on the right *y*‐axis by mean specificity score; (e) Network mapping of KO altered kinases in response to PE treatment (PKC isoforms) to an overlaid STIM1 node. Network mapping of identified upstream kinases and their relation to STIM1. Dark circle inset to the upper right of a protein indicates decreased signal in KO. Arrowheads denote literature annotated directions of interactions with the type of interaction indicated by arrow color (green‐positive; red‐negative). Legend is located on the right‐hand side of the figure panel. STIM1, stromal interaction molecule 1

It is well established that PE treatment promotes rapid and robust increases in the phosphorylation and/or dephosphorylation of several proteins such as ERK1/2, NFAT, PKC isoforms, and calcineurin; resulting in cardiac hypertrophic signaling (Liu et al., 2016; van Berio et al., 2011). PE also has been shown to promote hypertrophic signaling via the activation of SOCE (Hunton et al., 2002) and reduced expression of STIM1 and Orai1 protein was shown to reduce PE‐induced hypertrophy (Hulot et al., 2011; Voelkers et al., 2010). Therefore, we performed additional kinomic analysis of hearts from control and ^cr^STIM1^−/−^ mice treated with PE (15 min treatment; 15 mg/kg; s.c.). We first examined global changes in peptide phosphorylation in control and ^cr^STIM1^−/−^ hearts in response to saline and PE using heatmap analyses (Figure 2a). The heatmap shows the top 75 differentially regulated peptide targets between genotypes and in response to PE. The heatmap shows that overall peptide phosphorylation is reduced in the hearts of KO mice compared to control hearts at baseline (i.e., in response to saline) and that this further decreased in KO hearts following PE treatment. In addition, the heatmap also shows that in control hearts that phosphorylation of many peptides is somewhat reduced from control to PE, but this is increased in comparison to KO hearts at baseline. Furthermore, in control hearts PE‐mediated reductions in the phosphorylation of peptides derived from p53, VASP, CDN1A, and CENPA, all of which were increased in KO hearts. Figure 2b shows the phosphorylation time course for selected peptides that were differentially altered in control and ^cr^STIM1^−/−^ hearts in response to PE treatment. The phosphorylation time course showed that four peptides were preferentially inhibited by PE treatment in KO hearts. These peptides included the beta‐2 adrenergic receptor (ADBR2), glutamate ionotropic receptor kainate subunit 2 (GRIK2), protein tyrosine kinase 6 (PTK6), and vitronectin (VTNC). Next, we performed upstream kinase analysis to determine which kinases were giving rise to these changes in peptide phosphorylation in response to PE. In control hearts, following PE treatment we found significant, yet predicted reductions in the activity of CDK 14 (also known as PFTAIRE1 and PFTK1), AMPKα1, PKA, p70SK6, AKT1/2, protein kinase X‐linked (PRKX), AMP‐activated protein kinase family member 5 (nuak1, also known as ARK5), death‐associated protein kinase 3 (DAPK3), and CK2α (Figure 2c).

Next, we examined the effect of PE on kinase activity in ^cr^STIM1^−/−^ hearts (Figure 2d). We found that significant reductions in several PKC isoforms (i.e., PKCα, γ, δ,), PKAα, PKG1, PKG2, PRKX, CDK 17 (PCTAIRE2, also known as CDK17), and p70S6K in ^cr^STIM1^−/−^ hearts treated with PE compared to saline‐treated ^cr^STIM1^−/−^ hearts. The decreases in PKA, PRKX, and p70S6K in response to PE seem to be consistent between genotypes and likely reflect changes induced by PE that are not impacted by STIM1. In addition, the decreases in CK2α, AKT1/2, DAPK3, Nuak1, PCTAIRE2, and AMPKα seen in control hearts treated with PE do not seem to be changed in ^cr^STIM1^−/−^ hearts treated with PE, which may reflect the effect of loss of STIM1 in how these signaling pathways respond to PE. To investigate the impact of these changes in PKC further, we used network analysis to map the kinases altered in ^cr^STIM1^−/−^ hearts in response to PE to STIM1. Since PKC isoforms were greatly impacted in ^cr^STIM1^−/−^ hearts in response to PE, we focused on mapping PKC to STIM1 (Figure 2e), which reveals specific interactions between ERK1/2, and STIM1 and shows interactions between STIM1 with Ca^2+^ channels, cytosolic Ca^2+^ levels, and PKC isoforms. Collectively, these data suggest that STIM1 contributes to the cardiomyocyte responsiveness to PE through PKC‐dependent signaling.

Utilizing BioNavigator software, we performed additional analysis of the serine/threonine kinome array to identify any potential downstream altered peptides. Table 1 shows the downstream altered peptide targets in ^cr^STIM1^−/−^ hearts; many of which can have a significant impact on cardiac electrophysiology and contractility, including the L‐type Ca^2+^ channel (LTCC), delayed rectifier K^+^ channels, and Na^+^ channels in addition to PKC, PKA, and CaMKII. In addition, in Table 2, analyses showed changes in specific downstream altered peptides which have significant roles in regulating cardiac metabolism, such as HSL, which is consistent with our earlier study demonstrating the potential role of STIM1 in regulating cardiac fatty acid metabolism (Collins et al., 2018).

**TABLE 1:** Identified downstream peptide targets in ^cr^STIM1^−/−^ hearts

Bio navigator Ref #	Description
ACM5_498_510	Muscarinic acetylcholine receptor M5
ADRB2_338_350	Beta‐2 adrenergic receptor
CAC1C_1974_1986	Voltage‐dependent L‐type calcium channel subunit alpha‐1C (Cav1.2)
KAP2_92_104	cAMP‐dependent protein kinase type II‐alpha regulatory subunit
KAP3_107_119	cAMP‐dependent protein kinase type II‐beta regulatory subunit
KCC2G_278_289	Calcium/ calmodulin‐dependent protein kinase type II gamma chain
KCNA2_442_454	Potassium voltage‐gated channel subfamily A member 2 (subunit Kv1.2)
KCNA6_504_516	Potassium voltage‐gated channel subfamily A member 6 (subunit Kv1.6)
KPCB_19_31_A25S	Protein kinase C beta type
MYPC3_268_280	Myosin binding protein C, cardiac‐type (cardiac MyBP‐C_)
NOS3_1171_1183	Nitric oxide synthase, endothelial
PLM_76_88	Phospholemman precursor (FXYD domain‐containing ion transport regulator 1)
SCN7A_898_910	Sodium channel protein type 7 subunit alpha

Abbreviation: STIM1, stromal interaction molecule 1.

**TABLE 2:** Identified downstream metabolic peptide targets in ^cr^STIM1^−/−^ hearts

Bio navigator Ref #	Description
BAD_112_124	Bcl2 antagonist of cell death (BAD) (Bcl‐2‐binding component 6) (Bcl‐XL/Bcl‐2‐associated death promoter) (Bcl‐2‐like 8 protein)
BAD_93_105	Bcl2 antagonist of cell death (BAD) (Bcl‐2‐binding component 6) (Bcl‐XL/Bcl‐2‐associated death promoter) (Bcl‐2‐like 8 protein)
F263_454_466	6‐phosphofructo‐2‐kinase/fructose‐2,6‐biphosphatase 3
FOXO3_25_37	Forkhead box protein O3 (Forkhead in rhabdomyosarcoma‐like 1) (AF6q21protein)
GPR6_349_361	Sphingosine 1‐phosphate receptor GPR6 (G‐protein coupled receptor 6)
GYS2_1_13	Glycogen [starch] synthase
H2B1B_27_40	Histone H2B type 1‐B (H2B.f) (H2B/f) (H2B.1)
K6PL_766_778	6‐phosphofructokinase, (Phosphofructokinase1) (Phosphohexokinase) (Phosphofructo‐1‐kinase isozyme B) (PFK‐B)
LIPS_944_956	Hormone‐sensitive lipase (HSL)

Abbreviation: STIM1, stromal interaction molecule 1.

### 3.2 Effect of saline and PE treatment on kinase and phosphatase signaling in the ^cr^STIM1^−/−^ heart

**FIGURE 3:**
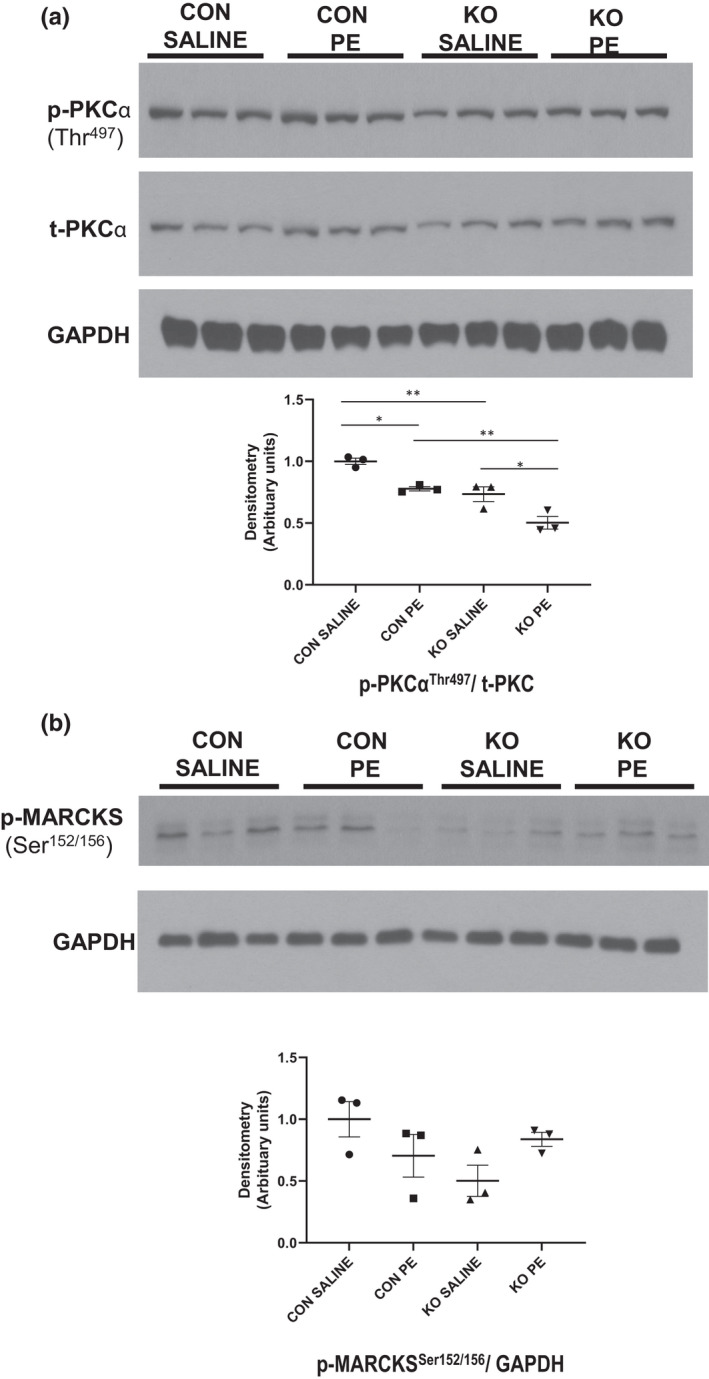
Decreased protein kinase C (PKC) isoforms in ^cr^STIM1^−/−^ hearts. Immunoblots and densitometric analysis of PKCα (a) and MARCKS (b) phosphorylation in hearts isolated from control and ^cr^STIM1^−/−^ mice treated subcutaneously with either 15 min saline or phenylephrine. Protein expression is normalized to total protein and/or GAPDH. STIM1, stromal interaction molecule 1. *p < 0.05, **p < 0.01, versus age‐matched control (ANOVA/Tukey's post hoc), *n* = 3 per genotype. STIM1, stromal interaction molecule 1

**FIGURE 4:**
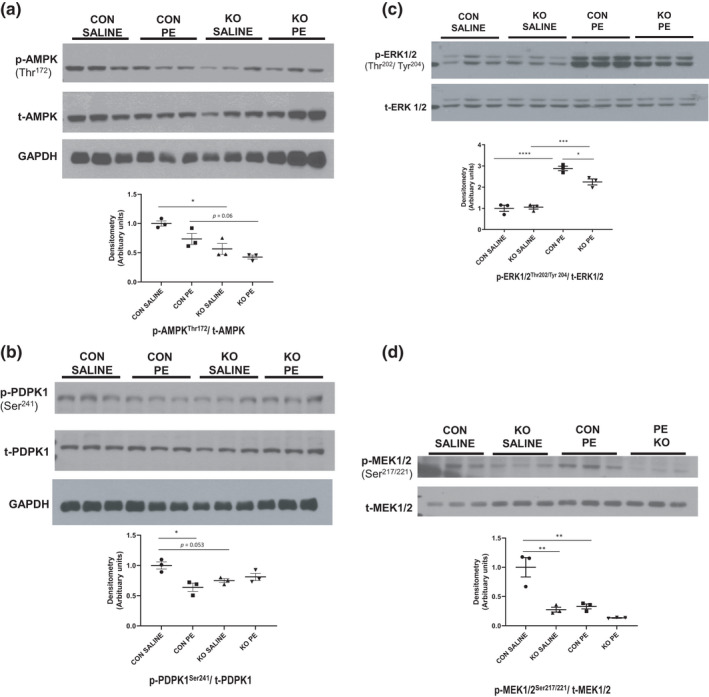
Decreased metabolic and growth kinases in ^cr^STIM1^−/−^ hearts. Immunoblots and densitometric analysis of AMPK (a), PDPK1 (b), ERK1/2 (c), and MEK1/2 (d), phosphorylation in hearts isolated from control and ^cr^STIM1^−/−^ mice treated subcutaneously with 15 min saline or phenylephrine. Protein expression is normalized to total protein and/or GAPDH. STIM1, stromal interaction molecule 1. *p < 0.05, **p < 0.01, ***p < 0.001, ****p < 0.0001, versus age‐matched control (ANOVA/ Tukey's post hoc), *n* = 3 per genotype

To validate the decrease in the activities of kinases in ^cr^STIM1^−/−^ hearts, we examined phosphorylation levels and whole‐cell protein levels of several kinome‐identified targets in control and ^cr^STIM1^−/−^ hearts following saline and PE treatment. We first examined PKC, which was identified through kinomic analyses to be reduced in ^cr^STIM1^−/−^ hearts in response to PE, and is a key player in Ca^2+^ signaling. Analysis of the Ca^2+^‐dependent isoform of PKC, PKCα, showed that at baseline (i.e., in response to saline) there was a marked reduction in the ratio of phosphorylated PKCα to total PKCα in ^cr^STIM1^−/−^ hearts versus control hearts (Figure 3a). In addition, in response to PE the ratio of phosphorylated PKCα to total PKCα was reduced in both control and ^cr^STIM1^−/−^ hearts (Figure 3a). These data reflect an overall reduction in PKCα phosphorylation in KO hearts and validate the signal identified in the kinomic array (Figure 3a). To further validate the documented reduction in PKCα‐dependent signaling in ^cr^STIM1^−/−^ hearts, we examined the phosphorylation of MARCKS at Ser152/156, a downstream target of PKC. There was a trend for a decrease in the phosphorylation of MARCKS at Ser152/156 in ^cr^STIM1^−/−^ hearts in comparison to control hearts in response to saline treatment (Figure 3b) (ANOVA *p* = 0.1058; Student's *t*‐test *p* = 0.0594). Immunoblot analysis did not reveal significant changes in the phosphorylation of MARCKS between genotypes in response to PE. Kinomic analysis also revealed that several metabolic kinases such as PDPK1 and AMPK, that scored “mid‐range” values during kinomic analysis, were reduced in the ^cr^STIM1^−/−^ hearts at baseline. Immunoblotting confirmed significant reductions in the phosphorylation of AMPK at Thr172 in ^cr^STIM1^−/−^ hearts versus control hearts at baseline (Figure 4a). There was also a trend (*p* = 0.06) for a decrease in the phosphorylation of AMPK in ^cr^STIM1^−/−^ hearts compared with control hearts following PE treatment. In addition, we observed significant reduction in the phosphorylation of PDPK1 at Ser241 in ^cr^STIM1^−/−^ hearts in response to saline treatment (Figure 4b). Furthermore, we observed a significant reduction in the phosphorylation of PDPK1 in control hearts between baseline and PE which was not observed in the hearts of KO mice (Figure 4b). Given that PE is known to increase the phosphorylation of ERK1/2 and that STIM1 is a key component of PE‐mediated signaling, we next examined the phosphorylation of both ERK1/2 and its upstream kinase, MEK1/2 in control and ^cr^STIM1^−/−^ hearts in response to saline and PE. There appeared to be no significant differences between genotypes at baseline with respect to ERK1/2 phosphorylation. As expected, there was a robust increase in the phosphorylation of ERK1/2 (Figure 4c) in control hearts response to PE. This increase in ERK1/2 phosphorylation was significantly reduced in the hearts of KO mice in response to PE; indicating a reduction in ERK1/2‐mediated signaling in KO hearts. Next, we looked at MEK1/2 phosphorylation in control and ^cr^STIM1^−/−^ hearts in response to saline and PE treatment (Figure 4d). Paradoxically, we found a significant reduction in MEK1/2 phosphorylation in control hearts treated with PE in comparison to those treated with saline. Interestingly, we observed that phosphorylation of MEK1/2 was reduced in ^cr^STIM1^−/−^ in comparison to control hearts with both saline and PE treatment. The reduction in MEK1/2 phosphorylation in KO hearts at baseline was comparable to the levels seen in control hearts treated with PE. Together, these data validate the findings from the kinomic array and show that significant reductions in the phosphorylation of several Ca^2+^‐dependent kinases occur in the hearts of ^cr^STIM1^−/−^ mice both at baseline and in response to PE.

### 3.3 Reduced calcineurin activity in the ^cr^STIM1^−/−^ heart

**FIGURE 5:**
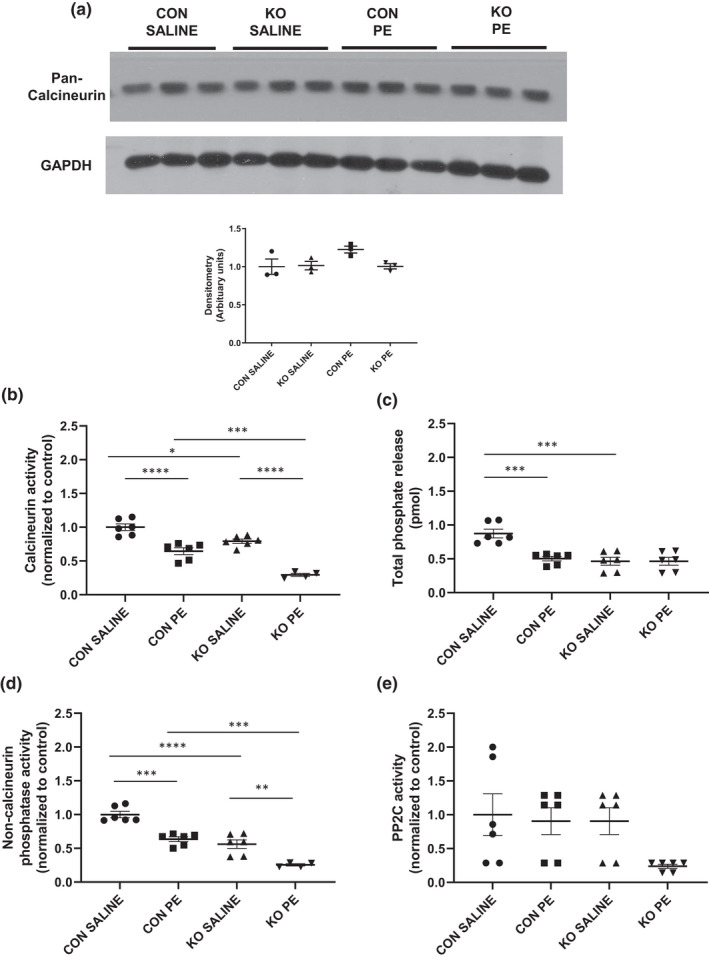
Reduced calcineurin activity in ^cr^STIM1^−/−^ hearts. (a) Immunoblots and densitometric analysis of calcineurin expression in hearts isolated from control and ^cr^STIM1^−/−^ mice treated with 15 min saline or phenylephrine (PE). Protein expression is normalized to GAPDH. *p* > 0.05, versus age‐matched control (ANOVA, Tukey's post hoc), *n* = 3 per genotype. Calcineurin and phosphatase activity was assayed in ^cr^STIM1^−/−^ and control heart lysates under saline and PE treatments showing the following parameters: (b) calcineurin activity (PP2B); (c) total phosphate released; (d) non‐calcineurin phosphatase activity (PP1, PP2A, and PP2C); and (e) PP2C phosphatase activity. STIM1, stromal interaction molecule 1. *p < 0.05, **p < 0.01, ***p < 0.001, ****p < 0.0001, versus age‐matched control (ANOVA/Tukey's post hoc), *n* = 6 per genotype

It is possible that in addition to kinase activity, calcium‐dependent phosphatase activity may be regulated by STIM1 and therefore altered in ^cr^STIM1^−/−^ hearts. Immunoblot analysis revealed that calcineurin protein expression was not significantly different between genotypes or in response to PE (Figure 5a). Interestingly, calcineurin activity was significantly reduced in ^cr^STIM1^−/−^ hearts in comparison to age‐matched littermate controls (Figure 5b) in both saline and PE‐treated groups. PE treatment resulted in a reduction in calcineurin activity with PE treatments in both genotypes. Total phosphatase activity, as reflected by total phosphate release (Figure 5c), was significantly decreased in KO hearts at baseline (i.e., in response to saline). PE treatment significantly reduced total phosphate release in control hearts but had no effect on phosphate release in KO hearts (Figure 5c). Non‐calcineurin activity (i.e., PP1 and PP2A) was significantly decreased in KO hearts versus control hearts in both saline and PE groups. There were no changes in PP2C activity (Figure 5e) in response to saline and PE in either genotype.

### 3.4 Effect of loss of STIM1 on the electrical activity of the heart under basal conditions and in response to PE

**FIGURE 6:**
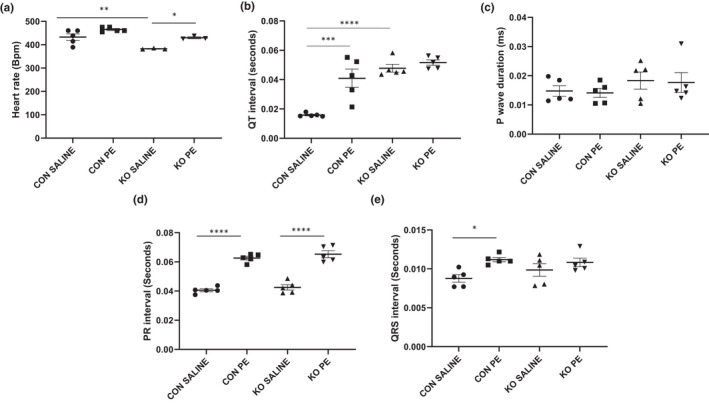
Electrocardiogram parameters in ^cr^STIM1^−/−^ hearts in response to saline and phenylephrine (PE) treatment. Analysis of electrocardiogram (ECG) parameters in control and ^cr^STIM1^−/−^ hearts treated with 15 min saline (baseline) or PE. (a) Heart rate; (b) QT interval; (c) P wave duration; (d) PR interval; and (e) QRS interval. STIM1, stromal interaction molecule 1. *p < 0.05, **p < 0.01, ***p < 0.001, ****p < 0.0001, versus age‐matched control (ANOVA/ Tukey's post hoc), *n* = 3–6 per genotype

Impaired PKC and PKG signaling have the potential to directly impact cardiomyocyte electrophysiology, which could impact the downstream physiological function of ^cr^STIM1^−/−^ hearts. We therefore, examined in vivo cardiac electrophysiology of control and ^cr^STIM1^−/−^ hearts under baseline and in response to PE treatment. ECG analysis revealed that heart rate was significantly reduced in ^cr^STIM1^−/−^ hearts at baseline (Figure 6a) and that there was also significant prolongation of QT interval (Figure 6b). We also observed no significant changes in P wave duration (Figure 6c) or amplitude (data not shown) between genotypes. PR interval, although increased with PE treatment, did not differ between genotypes (Figure 6d). In addition, there were no significant changes in QRS interval (Figure 6e) between genotypes at baseline or in response to PE treatment; however, PE treatment did increase QRS interval in control mice (i.e., saline vs. PE).

## 4 DISCUSSION

Several groups have shown that cardiomyocytes lacking or deficient in STIM1 develop a cardiomyopathy (Collins et al., 2014; Hulot et al., 2011; Luo et al., 2012; Ohba et al., 2009; Parks et al., 2016; Voelkers et al., 2010), and we have shown that loss of cardiomyocyte STIM1 is associated with the development of mitochondrial abnormalities (Collins et al., 2014, 2018), ER stress (Collins et al., 2014), and changes in cardiomyocyte glucose and lipid metabolism (Collins et al., 2018). Moreover, mice with cardiomyocyte over‐expression of STIM1 exhibit increased incidence of sudden cardiac death, and those that survive develop hypertrophy and heart failure (Correll et al., 2015). Therefore, it is increasingly evident that STIM1 plays an important role in regulating cardiac homeostasis. Surprisingly, despite these advancements, little is known of the specific signaling pathways that STIM1 regulates in the heart. Given its well‐documented canonical role in non‐cardiomyocyte cell types as an ER/SR Ca^2+^ sensor, we postulated that STIM1 may regulate the activity of Ca^2+^‐dependent kinases and phosphatases in the heart. Therefore, using our ^cr^STIM1^−/−^ mouse model we examined the effect of STIM1 deletion on the cardiac kinome and downstream physiological implications under basal conditions and the response to the α‐adrenergic agonist, PE. We found that loss of STIM1 in the heart is associated with an overall reduction in cardiomyocyte kinase and phosphatase activity, including specific reductions in the phosphorylation of several Ca^2+^‐dependent kinases, such as PKC, PKG, and AMPK, the latter consistent with our earlier study demonstrating that STIM1 has a regulatory role in cardiomyocyte metabolism (Collins et al., 2018). In addition, there were decreases in other Ca^2+^‐dependent kinases, such as PKCα and PKG which are known to regulate cardiac contractility. This was supported by alterations in the electrophysiological status of the heart and resulted in reduced responsiveness to PE.

Analysis of the serine/threonine kinome in control and ^cr^STIM1^−/−^ hearts showed that phosphorylation activity was decreased across multiple peptides in KO hearts, including targets of PKG 1 and 2 and several PKC isoforms. Several number of studies have linked PKC and PLC with the regulation of STIM1‐mediated Ca^2+^ entry (Gao et al., 2012; Kawasaki et al., 2010; Limnander et al., 2011; Yazbeck et al., 2017; Zheng et al., 2018); however, there is limited information linking STIM1 to the regulation of PKC or PLC. In acute lymphoblastic leukemia (ALL) cells, STIM1‐dependent Ca^2+^ entry has been linked to PKCβ2 activation (Zheng et al., 2018) and in vascular smooth muscle cells (VSMCs) STIM1 has been linked to increased PLCβ1 activity and subsequent phosphorylation of PKC (Shi et al., 2017). Moreover, in B‐cells STIM1 has also been associated with activation of non‐Ca^2+^‐dependent PKC isoforms, such as PKCδ (Limnander et al., 2011). These studies combined with our findings suggest that there may be reciprocal regulation between STIM1 and various PKC isoforms including non‐Ca^2+^‐dependent PKC isoforms, in different cell types including cardiomyocytes. The observation that non‐Ca^2+^‐dependent PKC isoforms were affected by the loss of STIM1 suggest additional regulatory mechanisms are at play that mediate the overall reduction in kinase activity in ^cr^STIM1^−/−^ hearts, such as changes in diacylglycerol levels. Given the multiple roles of different PKC isoforms in cardiomyocytes their potential regulation by STIM1 could be an important mechanism by which STIM1 maintains cardiomyocyte homeostasis. In addition, although Orai1 has been shown to be a downstream effector of PKG in human‐induced pluripotent stem cell‐derived cardiomyocytes (Wang et al., 2015), this is the first report to suggest that cardiomyocyte STIM1 may regulate PKG; however, little is known about the potential physiological role of STIM1 in regulating PKG signaling. PKG has wide‐ranging beneficial effects on cardiomyocyte function and impaired PKG signaling is increasingly associated with cardiac dysfunction (Oeing et al., 2020). Consequently, the observations here that STIM1 may contribute to PKG signaling has important implications for the role of STIM1 in cardiomyocytes. Given the key roles of both PKC and PKG in the heart, additional studies are warranted to elucidate the precise mechanisms by which STIM1 regulates the activity of these kinases.

Kinomic analysis also showed that AMPK phosphorylation was significantly reduced in ^cr^STIM1^−/−^ hearts. This is consistent with our previous findings that showed changes in glucose and lipid metabolism in ^cr^STIM1^−/−^ hearts and a reduction in AMPK phosphorylation (Collins et al., 2018). The identification of decreased AMPK phosphorylation in ^cr^STIM1^−/−^ hearts via two very different approaches strengthens the relationship between STIM1 regulation of AMPK in cardiomyocytes. In an osteosarcoma cell line, Mungai and colleagues reported that knockdown of STIM1 in the setting of hypoxia is associated with reduced AMPK phosphorylation (Mungai et al., 2011). Benard et al. examined the phospho‐kinome in HEK 293 cells treated with the SOCE activator, thapsigargin, and showed that the phosphorylation of several kinases including AMPK were reduced in cells treated with the STIM1/Orai1 blocker YM‐58483; however, they did not find changes in either PKC or PKG (Benard et al., 2016). In cardiomyocytes, they found that STIM1 silencing decreased AKT and GSK3β phosphorylation (Benard et al., 2016). In our earlier study, we found no changes in basal AKT or GSK3β phosphorylation in ^cr^STIM1^−/−^ hearts, but the insulin‐induced increase in AKT phosphorylation was attenuated (Collins et al., 2018). Although we did not identify AKT or GSK3β in our kinomics array, network analysis supports a possible link between STIM1 and GSK3β signaling (Figure 1c). Differences in kinases linked to STIM1 between our study and that by Benard et al. are inevitable given substantial methodological differences; however, both emphasize an important role for STIM1 in regulating a variety of different kinase signaling pathways. In addition, given the importance of the PI3K‐AKT‐mTOR pathway in cellular growth and survival pathways in the heart and the documented association of STIM1 with the activation of this pathway in other cell types (Zhou et al., 2017), additional studies are required to determine the relative importance of this association in the heart.

Our data show that MEK1/2 phosphorylation is reduced in ^cr^STIM1‐KO hearts at baseline and that ERK1/2 phosphorylation, although at similar levels between genotypes at baseline, is significantly reduced in response to PE to a greater extent in KO hearts versus control hearts. Interestingly, it has been previously shown in several studies from the Martin‐Romero lab that STIM1 phosphorylation plays a key effector role in the RAF‐MEK‐ERK pathway (Pozo‐Guisado et al., 2010, 2013; Pozo‐Guisado & Martin‐Romero, 2013; Tomas‐Martin et al., 2015); however, these studies have often used non‐cardiomyocyte cell types. It is somewhat puzzling that in our studies that PE led to a reduction in MEK1/2 phosphorylation but an increase in ERK1/2 phosphorylation. One would expect an increase in MEK1/2 phosphorylation to mediate an increase in ERK1/2 phosphorylation. This may suggest that the timing of our PE treatments may miss any PE‐induced increase in MEK1/2 phosphorylation prior to ERK1/2 phosphorylation or that ERK1/2 could be being regulated by additional kinases, such as PAK1 (Smith et al., 2008). In addition, it has been shown that ERK1/2 itself can feedback to inhibit both MEK1/2 and Raf (Lake et al., 2016; Liu et al., 2018). MEK1/2 activation is known to be affected by the presence of growth factors, cytokines, membrane depolarization, and Ca^2+^ influx so these factors could be contributing to the reduction in MEK1/2 phosphorylation in KO hearts. It will be essential to further examine the relationship between STIM1 and the MEK‐ERK signaling pathway using both pharmacological studies and studies activating and reducing identified kinases in the ^cr^STIM1^−/−^ hearts to determine the downstream effect on the STIM1‐KO phenotype. Consistent with a reduction in MEK‐ERK1/2 signaling in KO hearts, we also observe a reduction in calcineurin activity in KO hearts at baseline and in response to PE compared with control. Paradoxically, we observe a reduction in calcineurin activation with PE in control hearts. This could be due to fluctuations in Ca^2+^ levels, calmodulin levels, increased presence of endogenous calcineurin inhibitors (e.g., MCIP), or the timing of our PE treatments (i.e., 15min treatment). The Molkentin lab previously used acute PE treatment in wildtype mice (15 min) to show that phosphorylation of GATA4, ERK1/2, and p38 were all increased; however, in this study they did not examine calcineurin levels or activation (van Berlo et al., 2011). The same group has previously shown that ventricular calcineurin activation requires 24 h following hypertrophic stimuli (i.e., pressure‐overload; Lim et al., 2000); however, timing is likely different in response to acute PE treatment. Timing is likely the issue at play as it has been shown that calcineurin protein levels also increase with PE treatment (Lu et al., 2007), which we do not observe in our studies. Despite potential timing issues, our findings indicate that STIM1 plays a key role in the regulation of several Ca^2+^‐dependent kinases and phosphatases in the heart. Interestingly, it appears as though in some cases that PE treatment normalized the STIM1‐dependent effects in these studies which may suggest a role for STIM1 in α‐adrenergic signaling; however, additional studies are required to determine the mechanisms by which these changes are occurring. In addition, further studies determining whether the identified kinases are sensitive to SOCE or store depletion and determining whether kinase activity is sensitive to STIM1/SOCE inhibitors would provide additional insight on the specific mechanisms contributing to the activation of the identified kinases in ^cr^STIM1^−/−^ hearts. It is important to note, however, that the presence of SOCE in normal healthy adult cardiomyocytes remains a point of controversy, with some reporting that it is not present at all (Zhao et al., 2015), others showing it exists at very low but detectable levels (Hulot et al., 2011) and yet others suggesting STIM1 SOCE plays a specialized Ca^2+^ signaling role in coronary sinus cardiomyocytes (Zhang et al., 2020). Consequently, in future studies, it will be important to identify STIM1‐mediated kinase regulation that is potentially SOCE‐independent as well as those that are SOCE‐dependent.

Our downstream kinomic analyses identified several peptides targets in ^cr^STIM1^−/−^ hearts which included muscarinic acetylcholine receptor M5, β2 adrenergic receptor, LTCC, CaMKII, potassium channels (Kv1.2, Kv1.6), PKCβ, MYPC3, eNOS, phospholemman, and the sodium channel. The fact that CaMKII was identified as a potential target is consistent with earlier observations demonstrating that changes in cardiomyocyte STIM1 expression impact CaMKIIδ activation (Correll et al., 2015) and CaMKII phosphorylation (Zhang et al., 2015). On the other hand, the association between STIM1 and LTCC in cardiomyocytes identified by the kinomics analysis might seem surprising since the lack of STIM1 does not appear to have a direct effect on cardiac function. However, STIM1/SOCE have been shown to inhibit LTCC in HEK293 cells (Smith et al., 2008) and contribute to the regulation of LTCC in neuronal cells (Wang et al., 2010). More recently, Dittmer et al reported a glutamate‐dependent negative feedback regulation of LTCC by STIM1 (Dittmer et al., 2017). Thus, there is clear evidence for a role of STIM1 in regulating LTCC, which could have potential implications for cardiomyocyte Ca^2+^ signaling, and this may be reflected in the electrophysiological changes observed in the ^cr^STIM1^−/−^ heart ECG. Clearly, additional studies are required to understand the potential implications of STIM1‐mediated regulation of LTCC in cardiomyocytes.

Our kinomic validation relies heavily on phosphorylation site‐specific antibodies, which makes it difficult to assess the physiological relevance of the identified kinases and altered peptide targets. Future studies are needed to constitutively activate the identified kinases in the ^cr^STIM1^−/−^ mice to see if the present and previous (Collins et al., 2014, 2018) identified phenotypes of the ^cr^STIM1^−/−^ can be rescued will yield significant information. In a similar respect, future studies are also required to determine whether the specific kinase changes identified in our kinomic array result in changes in our ECG parameters. These studies would involve pharmacological activation or inhibition of the specific kinases to determine whether these mediate ECG changes in control mice and their impact in KO mice as if STIM1 is upstream of these kinases pharmacological treatment may prove unsuccessful.

The reduction in HR we observe in ^cr^STIM1^−/−^ hearts is consistent with studies from the Rosenberg lab in which hearts from mice lacking STIM1 had a significant reduction in HR at baseline at ECG, which they associated with defects in the sinoatrial node (Zhang et al., 2015). More recently, it has been shown that HR was between 10 and 60% lower in an inducible cardiomyocyte STIM1^−/−^ model versus littermate controls (Cacheux et al., 2019). In the latter study, they showed that prolonged cardiac action potential duration and spontaneous arrhythmogenic alternans occurred in ^cr^STIM1^−/−^ hearts, which was associated with increased mortality and likely supports a novel role of STIM1 in contributing to cardiac action potential dynamics. The heart rate reduction we observe in ^cr^STIM1^−/−^ hearts could reflect changes in PKC and PKG activity but could also reflect LTCC‐dependent and independent cellular remodeling. Future experiments are needed to interrogate the LTCC in the ^cr^STIM1^−/−^ hearts to determine the degree of interplay between STIM1‐LTCC in the heart but to also shed light on how the heart rate reduction is mediated in this model. In the same respect, studying the relevance of the kinomic changes on the physiology of additional Ca^2+^ handling proteins, such as RyR2 and PLN, is warranted. Our data also show that QT interval (QT) is prolonged in the hearts of ^cr^STIM1^−/−^ at baseline, which is of interest since long QT (LQT) is a precipitating factor in the development of arrhythmias. Of interest, a recent study identified a LQT syndrome associated with both changes in LTCC subunit CACNA1C and STIM1 (Tester & Ackerman, 2014). More specifically, the authors suggested that mutations in the STIM1 binding site on LTCC exist and disruption of the STIM1‐LTCC interaction, which usually reduced LTCC activity, could result in increases in LTCC and increases in action potential duration and prolonged QT interval. It is possible that the prolonged QT interval we observe is due to disruption of the association between LTCC and STIM1 and should be investigated further. Since we observe that the LTCC is an identified downstream peptide target of STIM1, this suggests that STIM1 may regulate LTCC to prevent arrhythmias, although additional studies are needed to examine. Furthermore, additional studies should be performed in conscious STIM1^−/−^ mice due to concerns associated with both the use of heart rate corrected QT interval and identification of the T wave in anaesthetized mice (Speerschneider & Thomsen, 2013). Zhang et al. (2020), have recently shown that cardiac STIM1 deletion resulted in slowed interatrial conduction and increased propensity of atrial arrhythmias and atrial fibrillation. The authors showed this was due to decreased STIM1‐mediated Ca^2+^ currents. Hypocalcemia (i.e., reduced calcium levels) has been linked to not only atrial fibrillation, but also *Torsades de pointes*, and ECG changes including QT prolongation and lengthened ST segment. It is possible that hypocalcemia may be contributing to the QT prolongation we observe in our ^cr^STIM1^−/−^ hearts; however, additional studies are required to determine both Ca^2+^ levels and cellular Ca^2+^ handling properties in ^cr^STIM1^−/−^ hearts. Of note, patients with Stormorken syndrome caused by a STIM1 mutation have been shown to exhibit hypocalcemia (Borsani et al., 2018). Hypokalemia (i.e., reduced potassium levels) has also been implicated in QT prolongation and changes in the ST segment through slowed conduction and delayed ventricular repolarization. It is possible that hypokalemia could contribute since in ^cr^STIM1^−/−^ hearts we observe these same changes, and several potassium channels that mediate cardiac repolarization were identified as downstream peptide targets of STIM1 in the kinomic analysis. However, hypokalemia is often also associated with changes in the p wave, which we do not observe in our ^cr^STIM1^−/−^ hearts and is consistent with previous reports in mouse models of STIM1 deficiency (Ohba et al., 2017). In our previous study (Collins et al., 2014), we found that by 36‐weeks our ^cr^STIM1^−/−^ mice had established cardiomyopathy, and by 50‐weeks of age over half of our ^cr^STIM1^−/−^ mice had died suddenly, which could be the result of a cardiomyopathy‐induced arrhythmia; however, in this study, arrhythmias or mechanisms contributing to increased mortality were not examined. In the present study, we observe STIM1‐dependent changes in ECG which may contribute to arrhythmic activity, if prolonged. It is very likely due to the relationship of HR with SV and CO that the changes we have observed previously (Collins et al., 2014) with regard to cardiac function in ^cr^STIM1^−/−^ are likely affected by this change in HR. Given that heart rate and heart rate variability are controlled by the interaction of the sympathetic nervous system (SNS) and the parasympathetic nervous system (PNS), with the SNS increasing heart rate through catecholamine release and the PNS being responsible for slowing heart rate via the release of acetylcholine, it appears as though lack of STIM1 may impact this balance. This is likely the case since it has been shown that STIM1^−/−^ hearts have altered cholinergic responsiveness (Zhang et al., 2015). Interestingly, the muscarinic acetylcholine receptor was identified as a potential downstream peptide target in ^cr^STIM1^−/−^ hearts (Table 1). Of note, muscarinic acetylcholine receptors have been previously associated with STIM1/Orai1‐mediated SOCE in neuroblastoma cells (Olianas et al., 2018); however, whether this is also the case in the heart remains to be determined. Further investigations are required to elucidate the potential involvement of STIM1 in sympathetic and parasympathetic regulation of the heart.

While there is a growing appreciation for the role of STIM1 in cardiac pathologies, such as hypertrophy and arrhythmogenesis, our understanding of the physiological role of STIM1 in the heart remains limited. Previously, we have shown that STIM1 is essential for maintaining cardiomyocyte homeostasis (Collins et al., 2014) and likely plays a role in the regulation of ER stress (Collins et al., 2014), mitochondrial dynamics (Collins et al., 2014), and myocardial metabolism (Collins et al., 2018). Here, we have shown that loss of STIM1 in the heart downregulates Ca^2+^‐activated kinase activity and attenuates their responsiveness to the α‐adrenergic agonist, PE, and this is associated with changes in cardiac electrophysiology all of which may also contribute to the phenotypic changes we have previously observed in ^cr^STIM1^−/−^ hearts (Collins et al., 2014, 2018). As our model is a constitutive cardiomyocyte‐specific STIM1^−/−^, it is possible that compensatory changes could have already occurred in the identified kinases therefore future studies utilizing inducible KO strategies are needed. In this study, we focused on the serine/threonine kinome; future studies will be needed to assess the impact of STIM1 deletion on the tyrosine kinome to generate a more complete picture of the STIM1‐regulated cardiac kinome. In addition, due to the low sample size in some experiments (i.e., *n* = 3–6/group), some kinomic changes could be masked. Furthermore, future studies will be required to determine if these changes also exist in the female heart. While additional studies are clearly needed, our findings show that STIM1 may be a previously unrecognized regulator for Ca^2+^‐dependent kinases in the heart and this may be an important factor for uncovering its physiological role in the heart.

## CONFLICT OF INTEREST

The authors do not have any conflict of interest and/or disclosures to declare.

## AUTHOR CONTRIBUTIONS

Helen E. Collins, Adam R. Wende, and John C. Chatham study conception and design; Helen E. Collins and Joshua C. Anderson performed experiments and analyzed data; Helen E. Collins and Joshua C. Anderson prepared figures; Helen E. Collins, Joshua C. Anderson, Adam R. Wende, and John C. Chatham interpreted results; Helen E. Collins drafted manuscript; Helen E. Collins, Joshua C. Anderson, Adam R. Wende, and John C. Chatham edited, revised, and approved the manuscript.
